# Chronic exposure to a gambling-like schedule of reward predictive stimuli can promote sensitization to amphetamine in rats

**DOI:** 10.3389/fnbeh.2014.00036

**Published:** 2014-02-11

**Authors:** Martin Zack, Robert E. Featherstone, Sarah Mathewson, Paul J. Fletcher

**Affiliations:** ^1^Cognitive Psychopharmacology Laboratory, Neuroscience Department, Centre for Addiction and Mental HealthToronto, ON, Canada; ^2^Translational Neuroscience Program, Department of Psychiatry, School of Medicine, University of PennsylvaniaPhiladelphia, PA, USA; ^3^Biopsychology Section, Neuroscience Department, Centre for Addiction and Mental HealthToronto, ON, Canada

**Keywords:** pathological gambling, sensitization, amphetamine, dopamine, uncertainty

## Abstract

Addiction is considered to be a brain disease caused by chronic exposure to drugs. Sensitization of brain dopamine (DA) systems partly mediates this effect. Pathological gambling (PG) is considered to be a behavioral addiction. Therefore, PG may be caused by chronic exposure to gambling. Identifying a gambling-induced sensitization of DA systems would support this possibility. Gambling rewards evoke DA release. One episode of slot machine play shifts the DA response from reward delivery to onset of cues (spinning reels) for reward, in line with temporal difference learning principles. Thus, conditioned stimuli (CS) play a key role in DA responses to gambling. In primates, DA response to a CS is strongest when reward probability is 50%. Under this schedule the CS elicits an expectancy of reward but provides no information about whether it will occur on a given trial. During gambling, a 50% schedule should elicit maximal DA release. This closely matches reward frequency (46%) on a commercial slot machine. DA release can contribute to sensitization, especially for amphetamine. Chronic exposure to a CS that predicts reward 50% of the time could mimic this effect. We tested this hypothesis in three studies with rats. Animals received 15 × 45-min exposures to a CS that predicted reward with a probability of 0, 25, 50, 75, or 100%. The CS was a light; the reward was a 10% sucrose solution. After training, rats received a sensitizing regimen of five separate doses (1 mg/kg) of d-amphetamine. Lastly they received a 0.5 or 1 mg/kg amphetamine challenge prior to a 90-min locomotor activity test. In all three studies the 50% group displayed greater activity than the other groups in response to both challenge doses. Effect sizes were modest but consistent, as reflected by a significant group × rank association (ϕ = 0.986, *p* = 0.025). Chronic exposure to a gambling-like schedule of reward predictive stimuli can promote sensitization to amphetamine much like exposure to amphetamine itself.

## Introduction

Addiction has been characterized as a brain disease caused by chronic exposure to drugs of abuse (Leshner, 1997). Neuroplasticity is thought to mediate the effects of such exposure (Nestler, 2001). Sensitization of brain dopamine (DA) systems is a form of neuroplasticity implicated in hyper-reactivity to conditioned stimuli (CS) for drugs, and compulsive drug seeking (Robinson and Berridge, 2001). Sensitization has been operationally defined by increased DA release in response to a CS for reward and by increased locomotor response to pharmacological DA challenge (Robinson and Berridge, 1993; Pierce and Kalivas, 1997; Vanderschuren and Kalivas, 2000). Although sensitization is only one of many brain changes linked with addiction (cf. Robbins and Everitt, 1999; Koob and Le Moal, 2008), changes in presynaptic dopamine release have been suggested to represent common neuroadaptations involved in addiction-based drug-seeking (e.g., relapse), in that drugs that induce locomotor sensitization to opiate (e.g., morphine) or stimulant challenge (e.g., amphetamine), also cause reinstatement of extinguished operant responses for heroin or cocaine self-administration—an animal model of relapse (Vanderschuren et al., 1999). Evidence that incentive sensitization (increased value of drug reward) is most pronounced after initial exposure to addictive drugs further suggests that sensitization may be involved in the early stages of addiction as well (Vanderschuren and Pierce, 2010).

Pathological gambling (PG) has been described as a behavioral addiction and recently reclassified to the same category as substance dependence disorders in the 5th edition of the Diagnostic and Statistical Manual of Mental Disorders (Frascella et al., 2010; A.P.A., 2013). This implies that PG may be caused by chronic exposure to gambling-like activity, that common mechanisms may mediate the effects of gambling and drug exposure (Zack and Poulos, 2009; Leeman and Potenza, 2012); and that sensitization of brain DA pathways may be one important element of this process.

Clinical evidence indirectly supports this possibility: Using positron emission tomography (PET) Boileau and colleagues found that male PG subjects exhibit significantly greater striatal DA release in response to amphetamine (0.4 mg/kg) than healthy male controls (Boileau et al., 2013). Overall group differences were significant in the associative and somatosensory striatum. In the limbic striatum, which includes the nucleus accumbens, the groups did not differ. However, in PG subjects, DA release in the limbic striatum correlated directly with the severity of PG symptoms. These findings are consistent with sensitization of brain DA pathways in PG, but also suggest some important differences with human substance dependent individuals and with the classic animal model of amphetamine sensitization. Unlike PG subjects and animals exposed to low doses of amphetamine (cf. Robinson et al., 1982), humans with substance dependence consistently exhibit decreased DA release to a stimulant challenge (Volkow et al., 1997; Martinez et al., 2007), and evidence from animals suggests that this may reflect deficits in DA function during the initial stages of abstinence following binge patterns of substance abuse (Mateo et al., 2005). In studies where stimulant sensitization is demonstrated in animals, enhanced DA release is usually observed in the limbic striatum rather than the dorsal (associative, somatosensory) striatum (Vezina, 2004). However, cue-induced (i.e., conditioned) drug-seeking in animals repeatedly exposed to cocaine has been linked with enhanced DA release in the dorsal striatum, a result thought to indicate a more habitual form of motivated behavior (Ito et al., 2002). Thus, the overall elevation in DA release in dorsal regions in PG subjects may be related to habit-based (inflexible, routinized) reward seeking involving “a progression from ventral to more dorsal domains of the striatum” (Everitt and Robbins, 2005, p. 1481), whereas the severity-dependent DA release in limbic striatum in these subjects may correspond more closely to incentive sensitization as typically modeled in animals. The PET findings cannot reveal whether DA hyper-reactivity was a pre-existing feature of these PG subjects, a consequence of gambling exposure, or a result of some other process entirely. To address this question, it is necessary to demonstrate induction of sensitization by chronic gambling exposure in subjects that are normal prior to exposure. This raises questions as to what features of gambling are most likely to induce sensitization.

Skinner noted that the variable schedule of reinforcement was fundamental to gambling's allure (or at least its persistence) (Skinner, 1953). Betting behavior in a slot machine game conforms well to the basic principles of instrumental conditioning, as reflected by a prospective correlation between monetary payoff and bet size on consecutive spins (Tremblay et al., 2011). Thus, variable ratio operant responding appears to provide an externally valid model of slot machine gambling.

Recent research with animals provides strong initial support for a causal effect of gambling exposure on sensitization. Singer and colleagues examined the effects of 55 1–h daily sessions of fixed (FR20) or variable (VR20) saccharin reinforcement in an operant lever-press paradigm on subsequent locomotor response to low dose (0.5 mg/kg) amphetamine in healthy male (Sprague Dawley) rats (Singer et al., 2012). They hypothesized that, if gambling leads to sensitization, rats exposed to the variable schedule, which mimics gambling, should exhibit greater response to amphetamine than rats exposed to the fixed schedule. As predicted, the VR20 group displayed 50% greater locomotor response to amphetamine than the FR20 group. In contrast, the groups displayed equivalent locomotion following a saline injection. These findings confirm that chronic exposure to variable reinforcement is sufficient to induce hyper-reactivity to a DA challenge in healthy animals randomized to the respective schedules.

A number of questions arise from this result: First, to what extent does the perceived contingency—or lack thereof—between the operant response and its outcome mediate these effects? In learning terms, does this effect involve a “response-outcome expectancy,” or might a similar effect be seen in the absence of an operant response, i.e., “a stimulus-outcome expectancy” in a Pavlovian paradigm (cf. Bolles, 1972)? Second, does the degree of contingency between the antecedent event (response or stimulus) and its outcome influence the degree of sensitization?

The second question concerns the role of uncertainty in sensitization. For example, do games whose outcome is truly random—completely unpredictable—have greater potential to induce sensitization than games where the odds of winning are clearly defined but not random, even if the absolute rate of reward is low? The present research addressed these questions.

The experimental design was informed by a seminal study on reward expectancy and DA neuron response in monkeys (Fiorillo et al., 2003). The animals in that study received a juice reward (US) under 0, 25, 50, 75, or 100% variable ratio schedules. The schedules were designated by 1 of 4 different CS (icons). The 0% schedule delivered reward as often as the 100% schedule, but omitted the CS. Firing rate of DA neurons during the interval between CS onset and US delivery or omission was the key dependent measure. The study found that DA response increased as a function of the uncertainty of reward delivery. Thus, under the 100% schedule the CS evoked little activity, under the 25 and 75% schedules, the CS evoked moderate and similar levels of activity, and under the 50% schedule the CS evoked maximal activity. In each case, firing rate escalated over the course of the CS-US interval, i.e., as the expectancy approached fruition.

These findings indicate that DA activity not only varies with whether or not reward is certain (Fixed Ratio) or uncertain (Variable Ratio), but also varies in inverse proportion to the amount of information about reward delivery conveyed by the CS. In the 100% condition, the CS evokes the reward expectancy and also perfectly predicts its delivery. In the 25 and 75% conditions, the CS evokes the expectancy and predicts reward delivery three out of four times. In the 50% condition the CS evokes the expectancy but provides no information about reward delivery beyond chance alone. Based on their findings, Fiorillo et al. concluded: “This uncertainty-induced increase in dopamine could contribute to the rewarding properties of gambling” (p. 1901).

The effects of 50% variable reward in a single session should not change over the course of multiple sessions because the likelihood of reward remains entirely unpredictable on every trial. Thus, when considering the conditions that would maximize chronic activation of DA neurons over repeated episodes of gambling the 50% schedule should engender the most enduring as well as the most robust effect. This is noteworthy given that the long run rate of reward (payoff > 0) observed over thousands of spins on a commercial slot machine was 45.8% (Tremblay et al., 2011). Thus, 50% variable reward appears to accurately reflect the payoff schedule administered by actual gambling devices.

The present study used the same conditioning schedules as Fiorillo et al. in a chronic exposure, between-groups' design with rats. Animals underwent ~3 weeks of daily conditioning sessions, where a CS (light) was paired with a US (small amount of sucrose). After the training phase, animals rested prior to assessment of sensitization indexed by locomotor response to amphetamine. Based on the literature, it was predicted that rats exposed to different reward schedules would not differ in their drug free locomotor behavior but would exhibit significantly different levels of locomotion following amphetamine, with the 50% group displaying a greater locomotor response to the drug relative to the other groups over the course of doses, a pattern that would be expected if the 50% animals had been previously exposed to additional doses of amphetamine itself (i.e., cross-sensitization).

## Experiment 1

### Materials and methods

#### Subjects

Four groups (*n* = 8/group) of adult (300–350 g) male Sprague-Dawley rats (Charles River, St. Constant, Quebec, Canada) were housed individually in clear polycarbonate boxes (20 × 43 × 22 cm) under a reverse 12:12 light-dark cycle. They received *ad libitum* access to food and water, and daily handling by an experimenter for 2 weeks prior to the study. Each group was conditioned under one of four variable reward schedules: 0, 25, 50, or 100%. The 75% group was omitted in this initial study, as Fiorillo et al. (2003) found equivalent post-CS DA release under 25 and 75% reward schedules, such that both conditions led to greater DA release than did the 100% CS-US condition, but less than the 50% condition.

#### Apparatus

Access to sucrose presentations and to the CS was provided individually in operant conditioning boxes (33 × 31 × 29 cm). Each box was equipped with a reinforcer magazine, located on the front wall. A light in the top of the magazine served as the CS. A motorized, solenoid-controlled liquid dipper could be elevated to the floor of the magazine. Events in the box were controlled by Med Associates equipment and software, using an in-house program written in MED-PC. Locomotor testing was conducted individually in Plexiglas cages (27 × 48 × 20 cm). Each cage was equipped with a monitoring system consisting of six photo-beam cells to detect horizontal movement.

#### Procedure

***Training***. The study was conducted in compliance with the ethical guidelines set out by the Canadian Council on Animal Care. Rats were food-restricted to 90% of their body weight for the duration of the study and housed individually. Each rat received 15 days of sucrose reward training (10% water solution at 0.06 ml per reward): 5 consecutive days × 3 weeks, with weekends off. Animals were maintained on standard chow before and after the training phase; sucrose exposure was restricted to the fifteen ~40-min training sessions. Each daily session consisted of 15 stimulus presentations (a light; CS), each separated by an inter-trial interval of 120 s. The light was located in the top panel of the magazine, and remained on for 25 s, with sucrose made available during the last 5 s. In the case of group 0 the sucrose dipper was raised every 140 s (for 5 s) but the stimulus light was not illuminated. This equated the interval between presentations of the dipper in group 0 and the other groups (120 + 25 s). Each treatment session lasted ~40 min. On average, group 25 received sucrose once for every four CS presentations; group 50 received sucrose once for every two CS presentations, and group 100 received sucrose after every CS presentation.

***Testing***. Two weeks after the last sucrose access (or “conditioning”) session, the locomotor response to d-amphetamine (AMPH; i.p.) was assessed. Rats were given three 2-h sessions to habituate to the locomotor boxes, followed by six AMPH test sessions. AMPH test days occurred at 1-wk intervals. On test days, rats were given 30 min to habituate to boxes then received a single 0.5 mg/kg dose of AMPH followed, on separate weekly sessions, by five 1.0 mg/kg doses (one dose per day) on test days 1 through 5. Post-AMPH locomotion was assessed for 90 min on each session.

#### Data analytic approach

Statistical analyses were conducted with SPSS (v. 16 and v. 21; SPSS Inc., Chicago IL). Immediate behavioral response to the CS was assessed in terms of nose pokes into the aperture where the sucrose was dispensed. The mean number of nose pokes during this interval (5 s per trial) was then compared to the mean number of nose pokes for the same duration (5 s) averaged over the time when the CS was absent. Group × Session ANOVAs of nose-pokes with CS present and absent tracked the acquisition of discriminative responding to the cue and indiscriminate nose poke responses under the different schedules over the course of the 15 sucrose training sessions.

Effects of treatment on locomotor responses were assessed with Group × Session ANOVAs for the drug-free habituation phase (three sessions), pre-sensitization 0.5 mg/kg AMPH challenge (one session), and during the five-session 1 mg/kg AMPH sensitization regimen, when groups were expected to differ in response to repeated doses of AMPH. Group × Session ANOVAs also assessed drug-free locomotor responses during the 30-min pre-injection habituation phase from each AMPH test session. Planned comparisons assessed the difference in mean performance for group 50 vs. group 0 (no expectancy control) and group 100 (no uncertainty control), by means of *t*-tests (Howell, 1992), using the MS error and df error terms for the relevant effect (i.e., group or group × session interaction) from the ANOVA (Winer, 1971). Polynomial trend analyses tested the profile of changes over the course of sessions.

To determine if approach responses in the presence and absence of the CS during the 15 sucrose training sessions contributed to variation in locomotor response to AMPH, or mediated group differences in AMPH response, follow-up analyses of covariance (ANCOVAs) were performed on the AMPH locomotor data, including total nose pokes (sum for 15 sessions) when the CS was absent as the covariate. A significant effect of the covariate would indicate that drug-free approach responses moderated (influenced the strength of) the effects of group or session. A decline in the significance of the effects of group or session in the presence of a significant covariate would indicate that approach responses mediated (accounted for) the effects of group or session. A decline in the significance of group or session effects in the absence of a significant covariate effect would simply reflect a loss of statistical power due to the reallocation of df from the error term to the covariate, and would not have bearing on the interpretation of the effects of group or session.

### Results

#### Nose pokes during sucrose conditioning sessions

**Figure 1:**
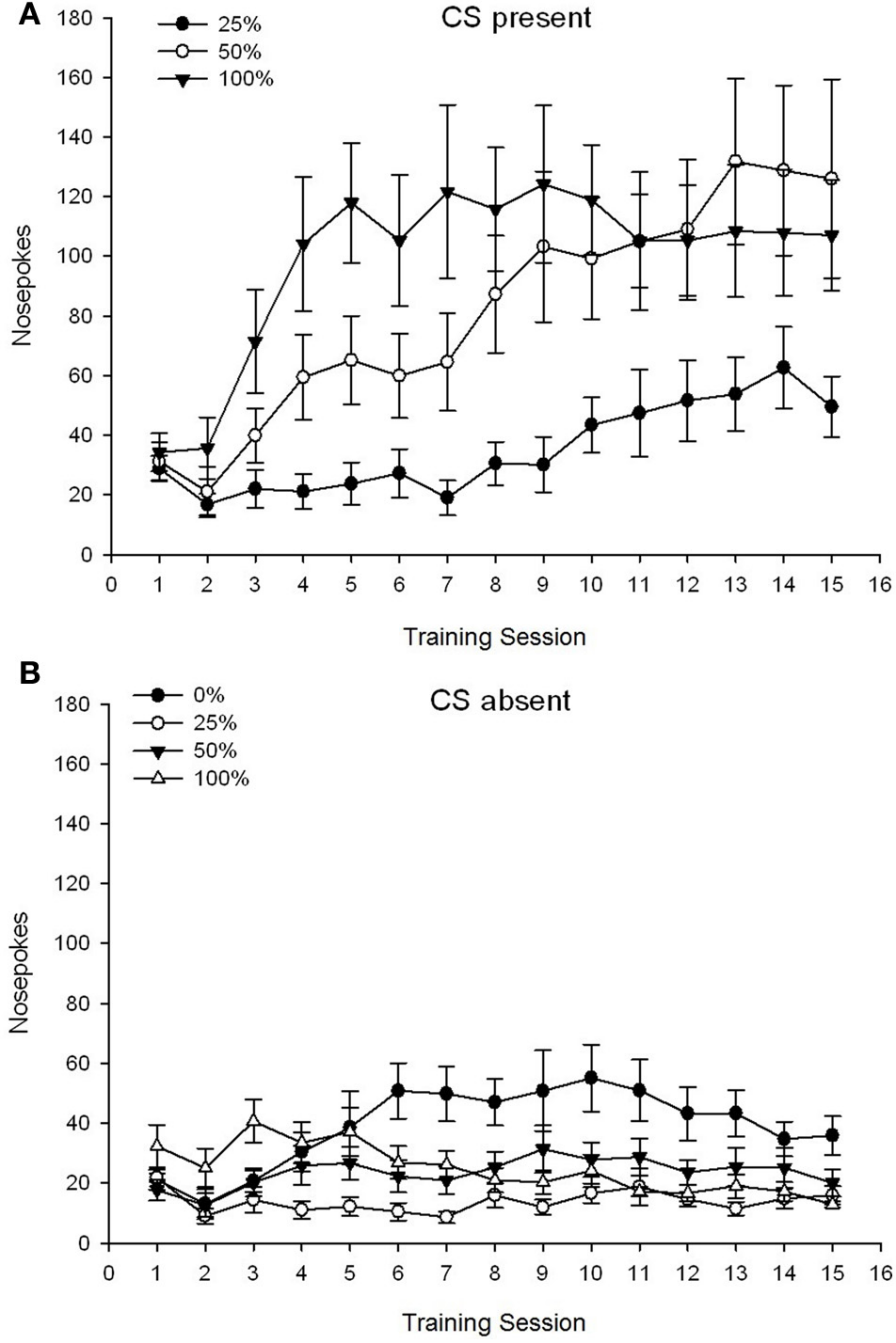
**Mean (SE) approach responses (nose pokes) on 15 sucrose training sessions in groups of Sprague Dawley rats (*n* = 8/group) exposed to sucrose reward (10% solution) delivered under 0, 25, 50, or 100% variable schedules**. The conditioned stimulus was a light (120 s). Group 0 received the same number of rewards as group 100 in the absence of conditioned stimuli. **(A)** Scores when CS was present (5 s × 15 trials). **(B)** Scores when CS was absent (average for 5 × 15 s while light was off).

***CS present***. Figure 1A shows the mean nose pokes for groups 25, 50, and 100 while the CS was present on the 15 sucrose conditioning sessions (nose pokes were not coded for group 0, which received no CS). A 3 Group × 15 Session ANOVA yielded significant main effects of Group, *F*_(2, 21)_ = 5.63, *p* = 0.011, and Session, *F*_(14, 294)_ = 14.00, *p* < 0.001, along with a significant Group × Session interaction, *F*_(28, 294)_ = 2.93, *p* < 0.001. Figure 1A indicates that the main effect of Session reflected an increase in nose pokes across sessions in all three groups, and the main effect of Group reflected generally higher overall scores in group 100 vs. group 25 with intermediate scores in group 50. A significant Group × Session interaction for the cubic trend, *F*_(2, 21)_ = 4.42, *p* = 0.030, indicated a rapid rise, dip, and leveling off in nose pokes over sessions in group 100, as against a linear increase over sessions in group 50, and a shallower linear increase over sessions in group 25.

***CS absent***. Figure 1B shows the mean nose pokes for all four groups for an equivalent duration (5 s × 15 trials) averaged over the time when the CS was absent. A 4 Group × 15 Session ANOVA yielded significant main effects of Group, *F*_(3, 28)_ = 7.06, *p* = 0.001, and Session *F*_(14, 392)_ = 2.84, *p* < 0.001, along with a significant Group × Session interaction, *F*_(42, 392)_ = 3.93, *p* < 0.001. A significant Group × Session interaction for the quadratic trend, *F*_(3, 28)_ = 3.91, *p* = 0.019, along with no interaction for the cubic trend, *F*_(3, 28)_ < 0.93, *p* > 0.44, reflected an “inverted-U” profile of nose pokes over sessions in group 0, as against a generally stable profile over sessions in the other groups.

#### Habituation to locomotor chambers

A 4 Group × 3 Session ANOVA yielded a main effect of Session, *F*_(2, 56)_ = 5.67, *p* = 0.006, and no other significant effects, *F*_(3, 28)_ < 1.60, *p* > 0.21. Mean (SE) beam breaks per 2 h in the locomotor boxes were 1681 (123) on session 1, 1525 (140) on session 2, and 1269 (96) on session 3. Planned comparisons found no significant differences between group 50 and group 0 or group 100 on the first or final habituation session, *t*_(84)_ < 1.69, *p* > 0.05. Thus, in the absence of AMPH, repeated exposure to the test boxes was associated with a consistent decline in spontaneous locomotor activity in the four groups (i.e., Session effect), and no differential response as a function of sucrose training schedule (no interaction).

#### Test sessions

***Effects of pre-sensitization 0.5 mg/kg AMPH challenge***.

*Pre-injection locomotion*. A 4 Group one-way ANOVA of locomotor response during the 30-min pre-injection habituation phase yielded no significant effects, *F*_(3, 28)_ < 1.05, *p* > 0.38. Planned comparisons found no significant difference between group 50 and group 0 or group 100, *t*_(32)_ < 0.87, *p* > 0.40. Therefore, baseline differences in pre-injection locomotion did not account for group differences in locomotor response to AMPH. Mean (SE) beam breaks for the sample were 559 (77).

*Post-injection locomotion vs. final drug-free habituation session*. A 4 Group × 2 Session ANOVA compared the groups' locomotor responses on the final habituation session, and immediately after the pre-sensitization 0.5 mg/kg AMPH challenge. Scores for the habituation session (120 min) were scaled to correspond with the duration of the AMPH test session (90 min) (raw habituation score × 90/120). The analysis yielded a significant main effect of Session, *F*_(1, 28)_ = 34.16, *p* < 0.001, and no other significant effects, *F*_(3, 28)_ < 2.26, *p* > 0.10. The Session effect reflected an increase in mean (SE) beam breaks in response to the dose, from 952 (72) to 1859 (151). Planned comparisons found no significant differences between group 50 and group 0 or group 100 in response to the dose, *t*_(56)_ < 1.72, *p* > 0.10. However, the rank order of beam break scores (M; SE) aligned with the hypothesis: group 50 (2205; 264) > group 0 (2025; 203) > group 100 (1909; 407) > group 25 (1296; 299).

***Effects of 1 mg/kg AMPH***.

*Pre-injection locomotion*. A 4 Group × 5 Session ANOVA of locomotor response during the 30-min pre-injection habituation phase on 1 mg/kg AMPH test sessions yielded a main effect of Session, *F*_(4, 112)_ = 43.64, *p* < 0.0001, and no other significant effects, *F*_(3, 28)_ < 0.97, *p* > 0.42. Planned comparisons found no significant difference between group 50 and group 0 or group 100 on the first or final test session, *t*_(140)_ < 0.84, *p* > 0.30. Therefore, baseline differences in locomotion did not account for group differences in locomotor response to AMPH. Mean (SE) beam break scores for the pre-dose habituation phase on sessions 1–5 were: 454 (30), 809 (53), 760 (36), 505 (35), 756 (39).

**Figure 2:**
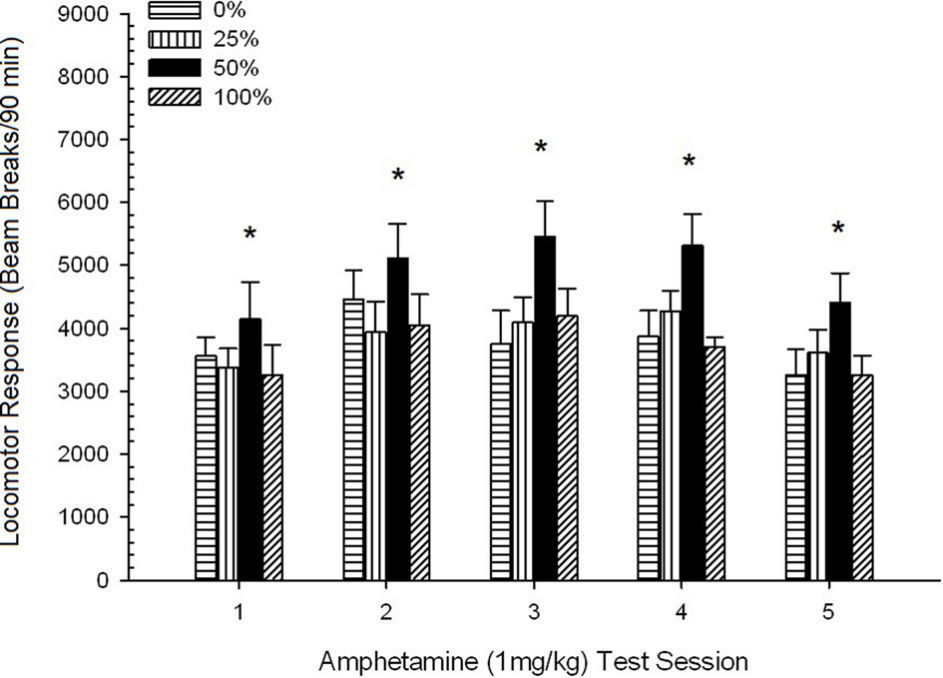
**Mean (SE) locomotor response (number of beam breaks in an electronic array per 90 min) to 1 mg/kg d-amphetamine (i.p.) on 5 weekly sessions in groups of Sprague Dawley rats (*n* = 8/group) previously exposed to 15 daily conditioning sessions with sucrose reward (10% solution) delivered under 0, 25, 50, or 100% variable schedules**. The conditioned stimulus was a light (120 s). Group 0 received the same number of rewards as group 100 in the absence of conditioned stimuli. ^*^p < 0.05 for mean difference between group 50 and group 0 as well as group 100, based on planned comparisons.

*Post-injection locomotion*. Figure 2 shows the effects of five injections of 1 mg/kg AMPH (one per week) on locomotor activity scores in the four groups. A 4 Group × 5 Session ANOVA yielded a main effect of Session, *F*_(4, 112)_ = 8.21, *p* < 0.001, a marginal main effect of Group, *F*_(2, 45)_ = 3.28, *p* = 0.085, and no significant interaction, *F*_(12, 122)_ < 0.77, *p* > 0.68.

Planned comparisons revealed that group 50 scores differed significantly from group 0, *t*_(14)_ = 2.19, *p* = 0.037, and group 100, *t*_(14)_ = 2.36, *p* = 0.025 [and differed marginally from group 25, *t*_(14)_ = 2.03, *p* = 0.051]. Thus, in group 50, locomotor response to 1 mg/kg AMPH reliably exceeded that of the other three groups across all five test sessions. Polynomial trend analysis detected a significant quadratic trend across sessions, *F*_(1, 28)_ = 32.47, *p* < 0.0001, and no other significant trends, *F*_(1, 28)_ < 1.78, *p* > 0.19. Figure 2 shows that this result reflected an “inverted U” pattern across sessions.

#### Control for variation in nose poke responding during sucrose training

The follow-up ANCOVA of locomotor responses to 1 mg/kg AMPH, with nose pokes (CS present) as the covariate, in the three groups that received the CS, yielded a marginal main effect of Group, *F*_(2, 20)_ = 3.07, *p* = 0.069, and no significant covariate-related effects, *F*_(4, 80)_ < 0.05, *p* > 0.85. Thus, cued approach responding during training did not explain significant variation in the locomotor response to 1 mg/kg AMPH in groups 25, 50, or 100.

The follow-up ANCOVA of locomotor responses to 1 mg/kg AMPH, with nose pokes (CS absent) as a covariate, yielded a significant effect of the covariate, *F*_(1, 27)_ = 6.17, *p* = 0.020, a significant main effect of Group, *F*_(3, 27)_ = 4.13, *p* = 0.016, a marginal Session × Covariate interaction, *p* = 0.080, and no other significant effects, *F*_(4, 108)_ < 1.48, *p* > 0.21. Thus, un-cued (indiscriminate) approach responding during training explained significant variation in locomotor response to 1 mg/kg AMPH. However, this variation was non-overlapping with group-related variance, because inclusion of the covariate in the analysis increased rather than decreased the significance of the group effect.

### Discussion

The nose poke data while the CS was present show that groups acquired the association between CS and sucrose delivery as reflected by an increase in cued responses over training sessions. The profile of responding over sessions while the CS was present suggested that 100 and 50% CS-US schedules were equally effective in eliciting approach, whereas the 25% schedule elicited a more modest increase in cue-induced approach. The nose poke data while the CS was absent suggest that groups that received any of the three CS-sucrose training schedules (group 25, 50, 100) rapidly learned to reduce their nose pokes in the absence of the CS, whereas animals in group 0, which received no CS, only learned to decrease their approach behavior to a limited degree after extensive training.

The habituation data show that the groups did not differ prior to AMPH and that repeated exposure to the test boxes was associated with decreased drug-free locomotor response. Therefore, between-group differences and increased responding over repeated doses of AMPH cannot be attributed to pre-existing differences in locomotor behavior.

Results of the pre-sensitization challenge with 0.5 mg/kg AMPH confirmed that the drug increased locomotor activity relative to the final drug-free habituation day. In line with the hypothesis, group 50 ranked higher than groups 0 or 100 (as well as group 25) in terms of mean response to the dose, although the mean differences between groups were not significant.

For the sensitization sessions, the between-groups' planned comparisons showed that prior exposure to 50% conditioned sucrose reward led to a significant increase in locomotor response to a 1.0 mg/kg dose of amphetamine relative to the other three schedules. This effect was evident from the first dose and did not change appreciably over repeated doses. The trend analysis indicated a biphasic response (for the full sample) to repeated doses of AMPH, increasing up to the third dose and decreasing thereafter. The results of the follow-up ANCOVA with nose-pokes (CS absent) as the covariate confirmed that differences in the four groups' locomotor responses to 1 mg/kg AMPH were not mediated by un-cued approach responding during the sucrose training sessions.

The group effect during the sensitization sessions is consistent with our hypothesis. The bi-phasic session effect is not consistent with the expected continued escalation in locomotor responses with repeated AMPH doses. This may be related to the dosing interval. To address this issue, a procedure (alternate daily doses) shown to induce consistent escalation in locomotor response to 1.0 mg/kg doses of AMPH (i.e., behavioral sensitization) should be employed. The impact of a sensitizing regimen of AMPH on subsequent response to a second 0.5 mg/kg challenge would further support the generality of this effect. Inclusion of a saline challenge prior to AMPH would determine the role of expectancy or injection-related (e.g., stress) effects on the locomotor response to AMPH. Inclusion of a 75% conditioned sucrose group would help to clarify the role of reward uncertainty vs. reward infrequency on the pattern of responses for groups 50 and group 25. In addition, to permit assessment (by ANCOVA) of the contribution of drug-free cued approach responses to locomotion under AMPH (using nose pokes with CS present as the covariate), nose pokes were also coded for group 0 during the interval when the CS was present in the other four groups (i.e., so that nose pokes from all five groups—including group 0 which received no CS—could be included in the analysis of covariance with CS present as the covariate). These refinements were incorporated in experiment 2.

## Experiment 2

### Materials and methods

The methodology of experiment 2 was similar to that of experiment 1 but revised to better approximate a regimen found to reliably induce AMPH sensitization (Fletcher et al., 2005). Changes were as follows: (a) The 75% CS-sucrose group (*n* = 8) was included; (b) During sucrose training, rats (except for group 0) received 20 CS (light) presentations (as opposed to 15 in experiment 1); (c) CS presentations were each separated by an average inter-trial interval of 90 s; range: 30–180 s (vs. 120 s in experiment 1), which offset the increase in training trials to equate the duration of each training session to that of experiment 1; (d) the duration of each of the three habituation sessions was decreased from 120 to 90 min to correspond with the duration of the test sessions; (e) A saline (i.p., 1 ml/kg) challenge (90 min) was added (post-sucrose training day 8), to assess the locomotor effects of injection *per se* (e.g., expectation, stress); (f) The 1 mg/kg sensitization sessions were held on alternate weekdays (post-training days 12–21) rather than at weekly intervals as in experiment 1; (g) Along with the pre-sensitization 0.5 mg/kg AMPH challenge (post-training day 9) a second post-sensitization 0.5 mg/kg AMPH challenge was added (post-sucrose training day 28), to test the generality of the sensitization effect across doses; (h) nose pokes while CS was present were coded for all groups (including group 0); (i) nose pokes while CS was absent were recorded specifically from the 5-s interval immediately prior to the onset of the CS to index premature approach responding.

### Results

#### Nose pokes during sucrose conditioning sessions

**Figure 3:**
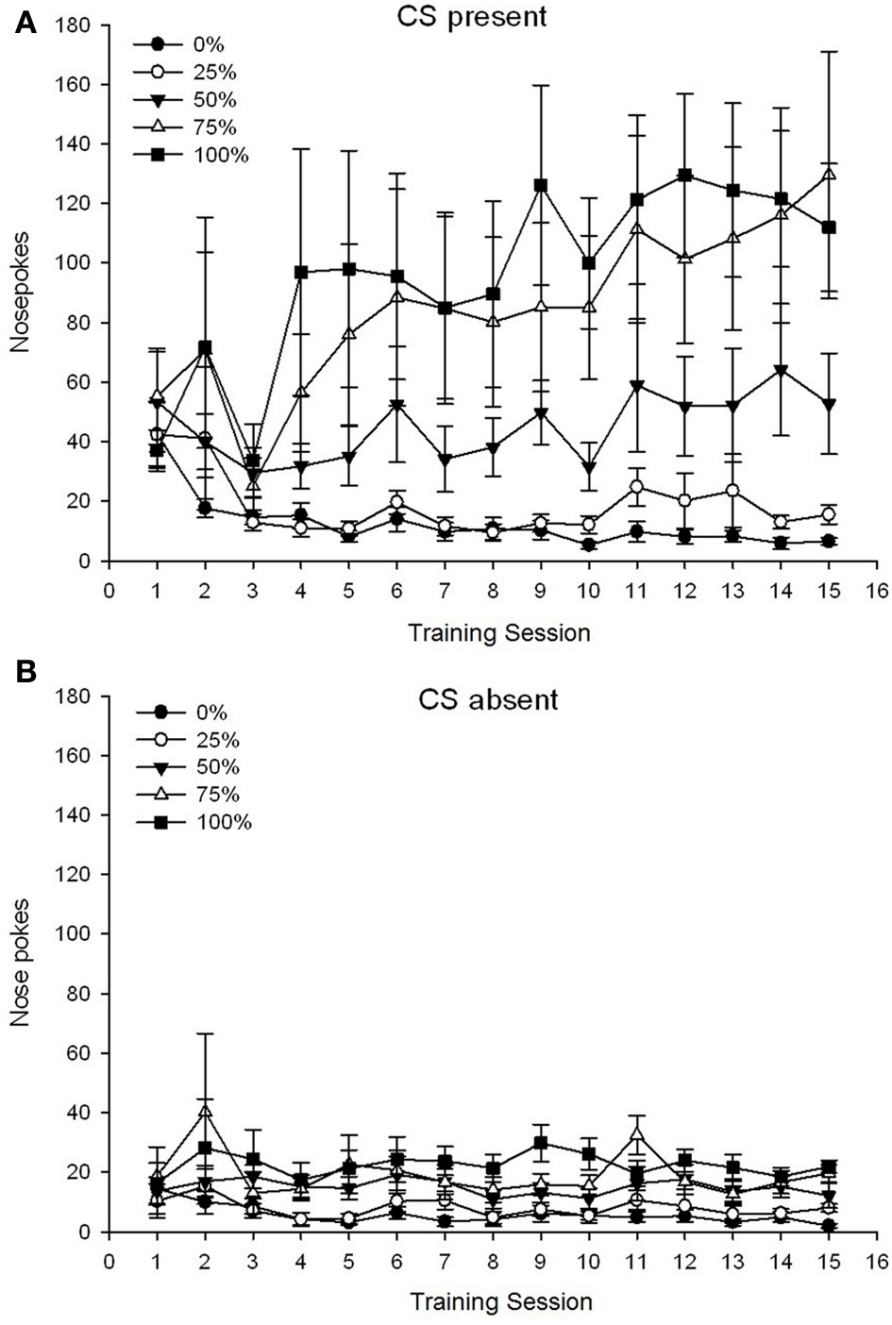
**Mean (SE) approach responses (nose pokes) on 15 sucrose training sessions in groups of Sprague Dawley rats (*n* = 8/group) exposed to sucrose reward (10% solution) delivered under 0, 25, 50, 75, or 100% variable schedules**. The conditioned stimulus was a light (120 s). Group 0 received the same number of rewards as group 100 in the absence of conditioned stimuli. **(A)** Scores when CS was present (5 s × 20 trials). **(B)** Scores when CS was absent (average for 5 × 20 s while light was off).

A 5 Group × 15 Session × 2 Phase (CS present, CS absent) ANOVA of nose pokes yielded significant main effects of Group, *F*_(4, 19)_ = 2.89, *p* = 0.050, Session *F*_(14, 266)_ = 2.28, *p* = 0.006, and Phase, *F*_(1, 19)_ = 14.72, *p* = 0.001, as well as a significant three-way interaction, *F*_(56, 266)_ = 1.38, *p* = 0.050. Panels **(A,B)** of Figure 3 plot the groups' mean nose poke scores for the CS present and CS absent phases, respectively. Comparison of the two panels reveals that the main effect of Phase reflected more overall nose poke responses when the CS was present vs. absent. Therefore, cued responses occurred significantly more often than did premature un-cued responses. The main effects of Group and Session were not readily interpreted due to the higher order interaction. This latter result reflected a convergence of scores for the five groups at a relatively stable low level across sessions when the CS was absent (Figure 3B), together with a divergence of scores into high (group 75, group 100), intermediate (group 50), and low (group 0, group 25) levels of nose poke responding over sessions when the CS was present (Figure 3A). Of the lower order polynomial trends (linear, quadratic, cubic) only the three-way interaction for the linear trend approached significance, *F*_(4, 19)_ = 2.32, *p* = 0.094, reflecting the generally monotonic increase in nose pokes over sessions in group 75 and relatively more rapid stabilization at high, intermediate, and low levels of responding in the other groups when the CS was present.

#### Habituation to locomotor boxes

A 5 Group × 3 Session ANOVA of drug-free locomotor responses yielded a significant main effect of Session, *F*_(2, 70)_ = 60.01, *p* < 0.0001, and no other significant effects, *F*_(4, 35)_ < 0.70, *p* > 0.60. Planned comparisons of group 50 with group 0 and with group 100 on the first and final habituation sessions yielded no significant effects, *t*'s < 0.84, *p* > 0.40. Therefore, mean drug-free locomotor response in the key groups did not differ prior to testing. Mean (SE) number of beam breaks per 90 min were 2162 (118) on session 1, 1470 (116) on session 2, and 1250 (98) on session 3.

#### Test sessions

**Figure 4:**
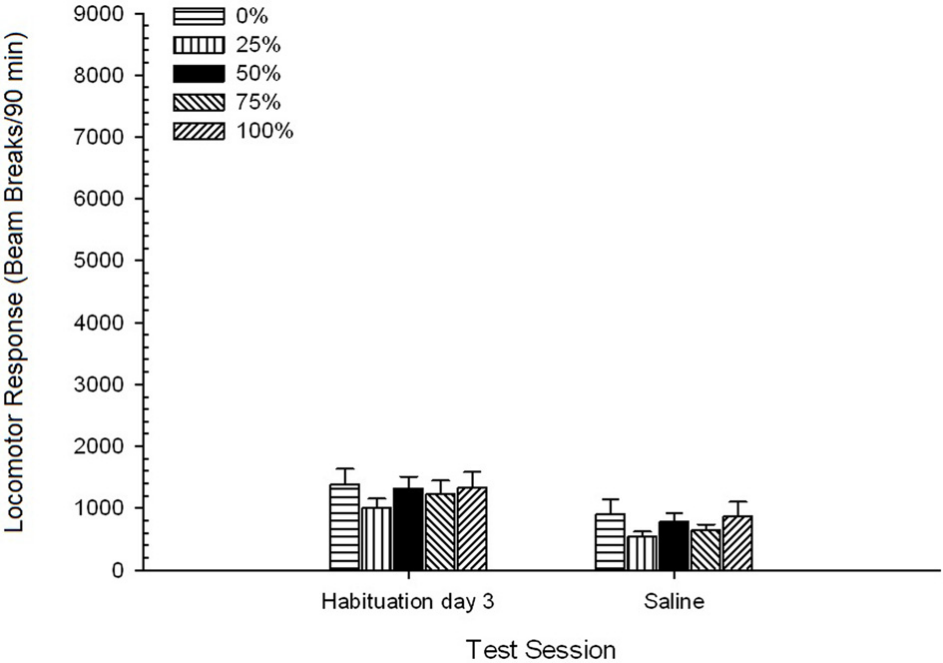
**Mean (SE) locomotor response (number of beam breaks in an electronic array per 90 min) on the last of 3 drug-free habituation sessions and on a subsequent session after saline injection (i.p., 1 ml/kg) in groups of Sprague Dawley rats (*n* = 8/group) previously exposed to 15 daily conditioning sessions with sucrose reward (10% solution) delivered under 0, 25, 50, 75, or 100% variable schedules**. The conditioned stimulus was a light (120 s). Group 0 received the same number of rewards as group 100 in the absence of conditioned stimuli.

***Saline***. A 5 Group × 2 Session ANOVA compared locomotor response on the final habituation session and saline challenge session. The ANOVA yielded a main effect of Session, *F*_(1, 35)_ = 62.46, *p* < 0.0001, and no other significant effects, *F*_(4, 35)_ < 0.65, *p* > 0.64. Figure 4 plots the group means and shows that the Session effect reflected an overall decrease in locomotor response from the final drug-free habituation session to the saline session, which did not vary by group. Thus, the decline in locomotor response seen over the three habituations sessions continued on the fourth drug-free exposure to the test boxes.

***Effects of 0.5 mg/kg AMPH***.

*Pre-injection locomotion*. A 5 Group × 2 Session ANOVA of pre-injection locomotion (30-min) on the pre- and post-sensitization 0.5 mg/kg AMPH test days yielded a significant main effect of Session, *F*_(1, 35)_ = 13.39, *p* = 0.001, and no other significant effects, *F*_(4, 35)_ < 1.79, *p* > 0.15. Planned comparisons found no significant differences between group 50 and group 0 or group 100 on the first session, *t*_(70)_ < 1.00, *p* > 0.30. However, on the second (post-sensitization) session group 50 (1203; 121) displayed significantly more pre-injection beam breaks (M; SE) than did group 100 (756; 103), *t*_(70)_ = 5.11, *p* < 0.001, but did not differ from group 0 (1126; 211), *t*_(7)_ < 0.88, *p* > 0.40. Therefore, baseline differences in locomotion did not account for group differences in locomotor response to the first 0.5 mg/kg dose of AMPH but may have contributed to differences between group 50 and group 100 in locomotor response to the second 0.5 mg/kg dose of AMPH. Mean (SE) beam breaks for the pre-injection phase on the first and second 0.5 mg/kg AMPH test sessions were 757 (41) and 974 (59).

**Figure 5:**
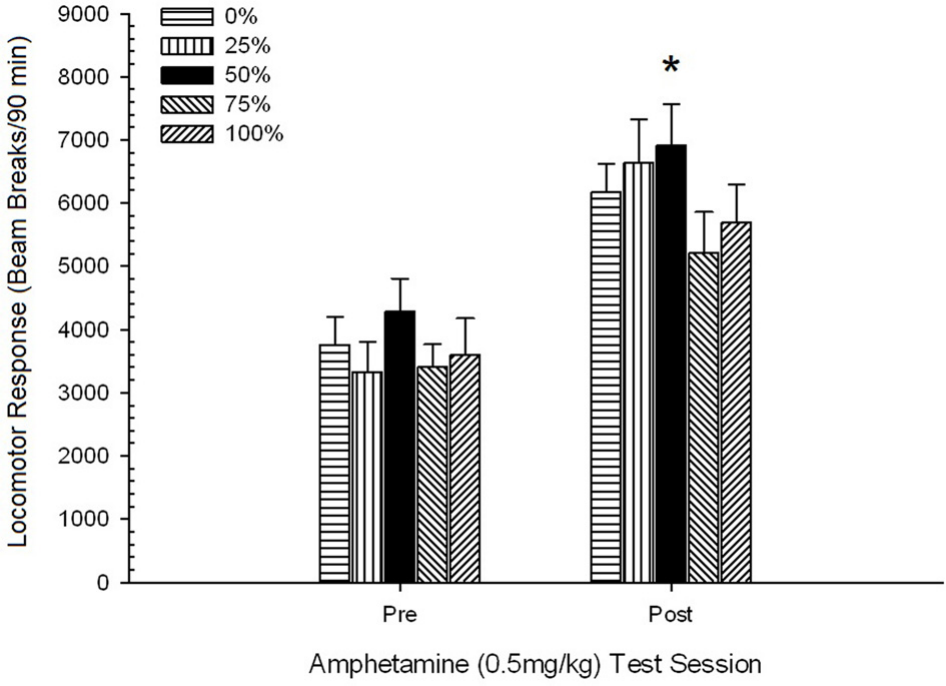
**Mean (SE) locomotor response (number of beam breaks in an electronic array per 90 min) to 0.5 mg/kg d-amphetamine on separate sessions before and after a 5-session sensitizing regimen of d-amphetamine (1.0 mg/kg; i.p. per session) in groups of Sprague Dawley rats (*n* = 8/group) previously exposed to 15 daily conditioning sessions with sucrose reward (10% solution) delivered under 0, 25, 50, 75, or 100% variable schedules**. The conditioned stimulus was a light (120 s). Group 0 received the same number of rewards as group 100 in the absence of conditioned stimuli. ^*^p < 0.05 for mean difference between group 50 and group 0 as well as group 100, based on planned comparisons.

*Post-injection locomotion*. A 5 Group × 2 Session ANOVA of locomotor response to 0.5 mg/kg AMPH before and after the 5-dose sensitizing regimen yielded a main effect of Session, *F*_(1, 35)_ = 76.05, *p* < 0.0001, and no other significant effects, *F*_(4, 35)_ < 1.10, *p* > 0.37. Figure 5 shows the mean scores for each group and session.

The figure shows that the Session effect involved a significant increase in overall mean (SE) beam breaks per 90 min from 0.5 mg/kg dose 1, 3674 (216) to 0.5 mg/kg dose 2, 6123 (275). The lack of interaction or group effect suggested that sensitization to AMPH did not vary reliably across groups. Despite the lack of significant group-related effects in the ANOVA, inspection of the figure reveals that group 50 displayed the greatest response to both the first and second 0.5 mg/kg doses. Planned comparisons of response to the first 0.5 mg/kg dose revealed no significant difference between group 50 and group 0 or group 100, *t*'s_(35)_ < 0.48, *p* > 0.50. However, in response to the second (post-sensitization) 0.5 mg/kg dose, group 50 displayed significantly greater locomotion than group 0, *t*_(35)_ = 2.00, *p* < 0.05, as well as group 100, *t*_(35)_ = 3.29, *p* < 0.01.

In light of the significant group difference in pre-injection locomotion on the second 0.5 mg/kg AMPH session reported above, a follow-up 5 Group × 2 Session ANCOVA of locomotor response to 0.5 mg/kg AMPH was conducted, controlling for pre-injection locomotion on the second session. This analysis yielded a significant effect of the covariate, *F*_(1, 34)_ = 8.65, *p* = 0.006, a main effect of Session *F*_(1, 34)_ = 10.83, *p* = 0.002, and no other significant effects, *F*_(4, 34)_ < 0.85, *p* > 0.50. Importantly, planned comparisons based on the MS error and df error from the ANCOVA confirmed that mean locomotor response to the second 0.5 mg/kg dose of AMPH remained significantly greater in group 50 than group 100, *t*_(34)_ = 3.09, *p* < 0.01, and group 0, *t*_(34)_ = 1.88, *p* < 0.05 (one-tailed), when pre-injection variation from session 2 was controlled. Thus, group 50 displayed significantly greater post-sensitization locomotor response to 0.5 mg/kg AMPH than did group 100 or group 0, and these group differences were not mediated by pre-injection locomotion on test days.

***Effects of 1.0 mg/kg AMPH***.

*Pre-injection locomotion*. A 5 Group × 5 Session ANOVA of 30-min pre-injection scores for the 1 mg/kg AMPH sensitization sessions yielded a main effect of Session, *F*_(4, 140)_ = 16.70, *p* < 0.0001, and no other significant effects, *F*_(4, 35)_ < 0.94, *p* > 0.45. Planned comparisons found no significant difference in pre-injection locomotion between group 50 and group 0 or group 100 on the first session, *t*_(175)_ < 1.66, *p* > 0.10. However, on the final session, group 50 (1167; 140) displayed significantly more beam breaks (M; SE) than did group 100 (1000; 99), *t*_(175)_ = 2.35, *p* < 0.05, but did not differ from group 0 (1085, 120), *t*_(175)_ < 1.16, *p* > 0.20. Therefore, differences in pre-injection locomotion contributed to differences between groups 50 and 100 in locomotor response to the final 1 mg/kg AMPH dose. Mean (SE) overall beam breaks for the sample during the pre-injection phase for Sessions 1 through 5 were: 810 (46), 784 (52), 760 (53), 726 (46), 1009 (51).

**Figure 6:**
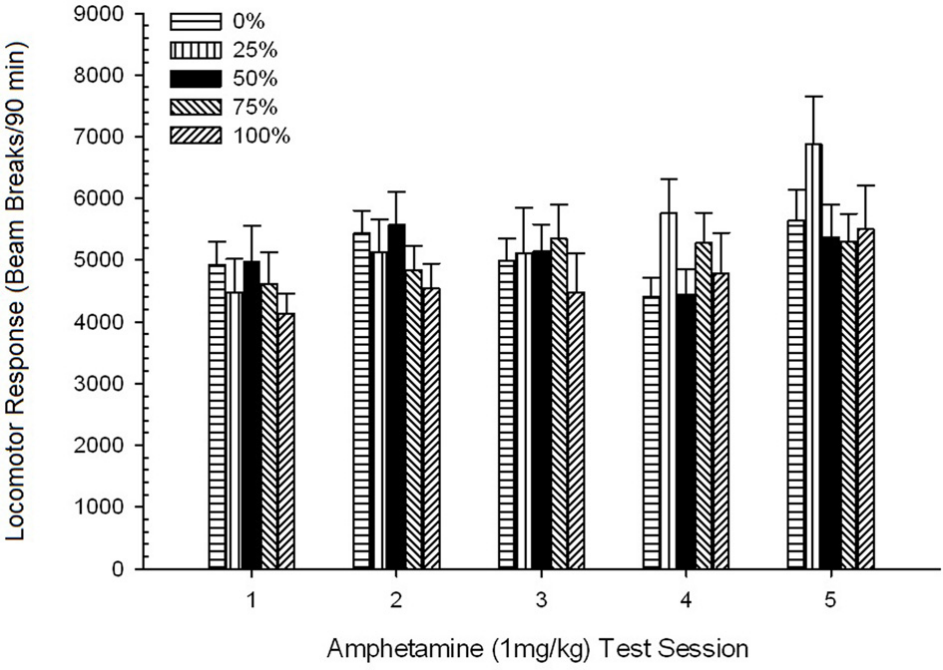
**Mean (SE) locomotor response (number of beam breaks in an electronic array per 90 min) to 1 mg/kg d-amphetamine (i.p.) on 5 weekly sessions in groups of Sprague Dawley rats (*n* = 8/ group) previously exposed to 15 daily conditioning sessions with sucrose reward (10% solution) delivered under 0, 25, 50, 75, or 100% variable schedules**. The conditioned stimulus was a light (120 s). Group 0 received the same number of rewards as group 100 in the absence of conditioned stimuli.

*Post-injection locomotion*. A 5 Group × 5 Session ANOVA of responses to 1 mg/kg AMPH yielded a significant main effect of Session, *F*_(4, 140)_ = 6.72, *p* < 0.001, a marginal Group × Session interaction, *F*_(16, 140)_ = 1.57, *p* = 0.085, and no main effect of Group, *F*_(4, 35)_ < 0.44, *p* > 0.77. Polynomial trend analyses revealed a significant linear trend, *F*_(1, 35)_ = 9.19, *p* = 0.005, and cubic trend, *F*_(1, 35)_ = 21.63, *p* < 0.001, over sessions 1 through 5. Figure 6 shows the mean locomotor scores for each group and session.

The figure shows that the Session effect reflected a significant increase in overall mean (SE) beam breaks for the full sample from session 1, 4624 (213) to session 5, 5736 (272), confirming the emergence of sensitization to AMPH. The cubic trend denoted relative maxima on sessions 1, 3, and 5, with dips on sessions 2 and 4, particularly for groups 0 and 50. The figure also reveals that, despite the lack of significant interaction, group 25 displayed progressively greater locomotor response over sessions and differed considerably from the other groups on sessions 4 and 5 (9 and 22% greater respectively, than next highest group). Planned comparisons found that group 50 did not differ significantly from groups 0 or 100, *t*_(175)_ < 0.89, *p* > 0.40 on the first or final 1 mg/kg AMPH test session.

#### Control for variation in nose poke responding during sucrose training

Two 5 Group × 2 Session ANCOVAs of locomotor response to 0.5 mg/kg AMPH before and after the sensitization regimen, including total nose pokes during sucrose training with CS present and with CS absent as separate covariates, found no significant effects for either covariate, *F*_(1, 18)_ < 1.03, *p* > 0.31. Therefore, approach responding during training did not mediate group differences in response to 0.5 mg/kg AMPH.

Two 5 Group × 5 Session ANCOVAs of locomotor response to 1 mg/kg during the sensitization sessions with total nose pokes (CS present, CS absent) as separate covariates yielded no significant effects of the covariate while the CS was present, *F*_(4, 104)_ < 1.04, *p* > 0.38, and a marginal main effect of the covariate while the CS was absent, *F*_(1, 18)_ = 3.32, *p* = 0.085.

### Discussion

The results of this study did not consistently support the hypothesis that group 50 would demonstrate higher locomotor response over sessions compared to the other groups. The 1 mg/kg AMPH data confirmed the emergence of sensitization with the alternate-day dosing regimen. The pattern across groups indicated a trend for greater sensitization during the latter sessions in group 25, with no such evidence for group 50. In contrast, the 0.5 mg/kg dose results indicated a trend for greater sensitization in group 50, while at the same time confirming a significant overall increase in locomotor response across groups to the second vs. the first 0.5 mg/kg AMPH dose. The null effect of saline injection confirmed that expectancy or injection-related stress did not contribute to the AMPH effects.

The nose poke data again revealed an overall increase in approach responding over the course of training sessions when the CS was present, with no corresponding increase when the CS was absent. Therefore, the animals appeared to acquire the association between the CS and the prospect of sucrose reward. Group differences in the frequency of nose pokes when the CS was present conformed roughly to the frequency of reward delivery under the respective schedules, with groups 75 and 100 displaying the most nose pokes, group 50 displaying intermediate numbers of nose pokes, and groups 0 and 25 displaying the fewest nose pokes. These results suggest that the CS came to control approach responding in a manner consistent with the overall probability of reward. Although speculative, one possible explanation for the lower nose poke rates with CS present in group 50 in experiment 2 vs. experiment 1 may be the shortening of the inter-trial interval, as longer inter-trial intervals (experiment 1) appear to encourage impulsive tendencies and this is associated with increased turnover of DA in anterior cingulate, prelimbic and infralimbic cortices (Dalley et al., 2002). Therefore, the 30% reduction in inter-trial interval in experiment 2 (and 3) may have altered cortical DA levels and promoted more selective (i.e., guided by the relative frequency of reward) vs. impulsive (not guided by reward frequency) approach responding in group 50 during training trials in experiment 2 as compared with experiment 1.

The lack of significant covariate-related effects for nose pokes in the CS present condition in the ANCOVAs indicates that approach responding during sucrose training did not mediate the effects of the different CS-sucrose schedules on responses to AMPH. The marginally significant effect of the covariate for the CS absent condition in the ANCOVA of locomotor responses to 1 mg/kg AMPH suggests that the tendency toward premature drug-free responding explained some of the variability in locomotor effects of AMPH during the sensitization sessions.

Together, the evidence suggests that the effects of conditioning history may be more discernible with 0.5 AMPH than with 1 mg/kg AMPH, and that a protocol that generates sensitization in the absence of any other manipulation may obscure or render redundant the effects of a putative sensitization-promoting behavioral manipulation (i.e., chronic variable reward).

Behavioral sensitization to AMPH is a robust effect in the laboratory. However, outside the laboratory, only a minority of individuals who gamble chronically escalate to pathological levels. Although risk for sensitization is related to risk for addiction (or drug seeking), especially for psychostimulants (Vezina, 2004; Flagel et al., 2008), many factors aside from sensitization risk may predispose one to addiction (e.g., Verdejo-Garcia et al., 2008; Conversano et al., 2012; Volkow et al., 2012). Nevertheless, trait factors that confer vulnerability to sensitization may interact with conditioning history to accentuate the effects of unpredictable reward (i.e., 50% CS-US schedule) on DA system reactivity. To investigate this possibility, experiment 3 employed the same procedure as experiment 2 but used Lewis strain instead of Sprague Dawley strain rats.

Sprague Dawley rats display intermediate levels of DA transporters, with lower levels than Wistar strain rats (Zamudio et al., 2005), but higher levels than Wistar-Kyoto rats (a “depressive”-like strain) in the nucleus accumbens, amygdala, ventral tegmental area and substantia nigra (Jiao et al., 2003). This profile may render Sprague Dawley rats only moderately sensitive to environmental or pharmacological manipulations of DA function. In contrast, Lewis rats exhibit low levels of DA transporters as well as D2 and D3 DA receptors in the nucleus accumbens and dorsal striatum compared to other strains (e.g., F344) (Flores et al., 1998). These morphological differences may contribute to Lewis rats' differential response to DA manipulations. Lewis rats also exhibit a range of accentuated responses to experimental drug manipulations compared to other strains (e.g., F344). Most importantly, Lewis rats display greater sensitization to methamphetamine, characterized by low response to initial doses but higher response to later doses (Camp et al., 1994). Lewis rats also exhibit greater locomotor sensitization to a range of doses of cocaine (Kosten et al., 1994; Haile et al., 2001). Based on this pattern of effects, we surmised that Lewis rats would enable us to investigate whether susceptibility to sensitization amplifies the effects of conditioning schedule on subsequent response to AMPH.

## Experiment 3

### Materials and methods

The methodology was the same as in experiment 2, aside from the use of Lewis rats (200–225 g on arrival, Charles River, Quebec, Canada).

### Results

#### Nose pokes during sucrose conditioning sessions

**Figure 7:**
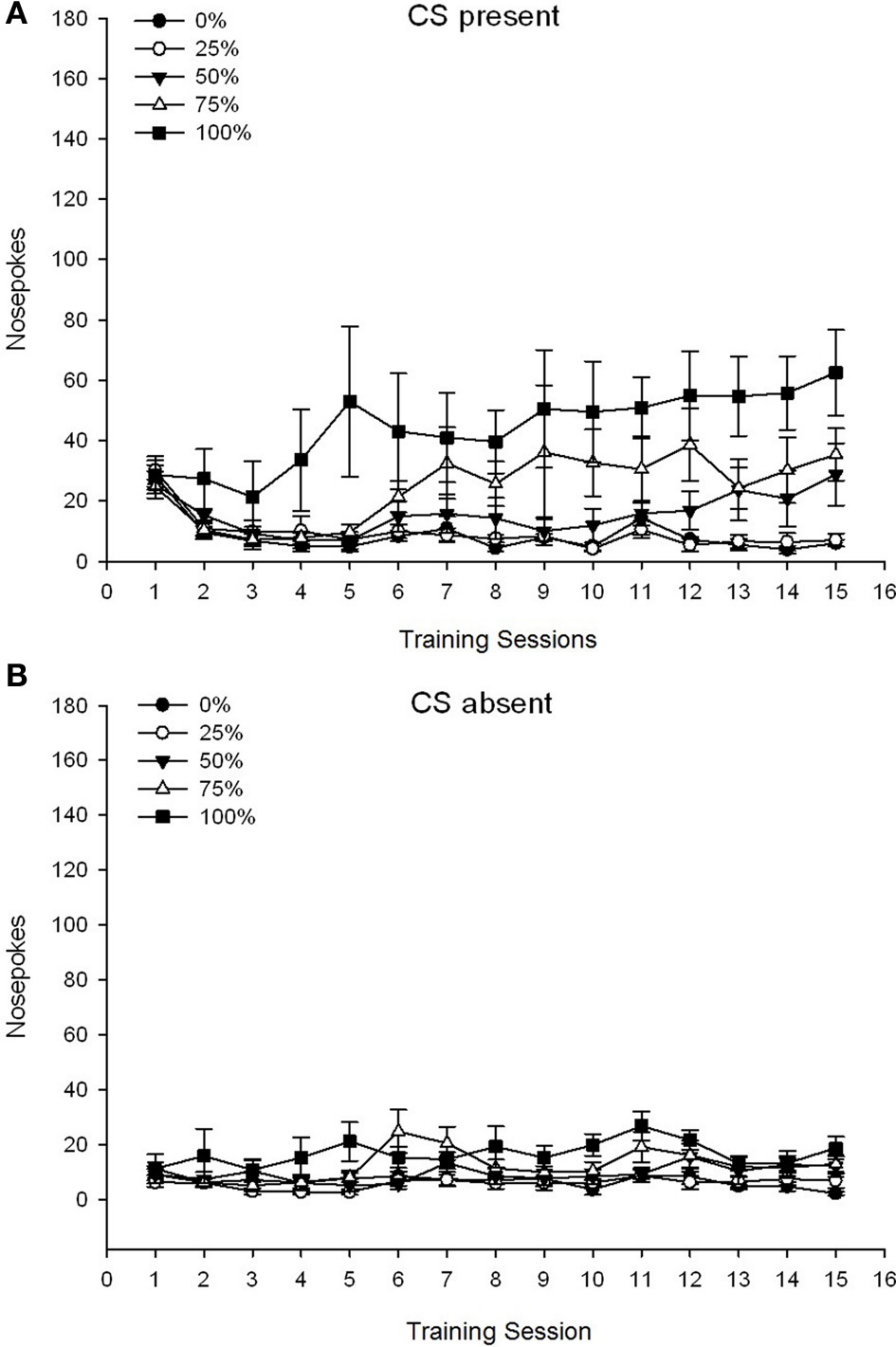
**Mean (SE) approach responses (nose pokes) on 15 sucrose training sessions in groups of Lewis rats (*n* = 8/group) exposed to sucrose reward (10% solution) delivered under 0, 25, 50, 75, or 100% variable schedules**. The conditioned stimulus was a light (120 s). Group 0 received the same number of rewards as group 100 in the absence of conditioned stimuli. **(A)** Scores when CS was present (5 s × 20 trials). **(B)** Scores when CS was absent (average for 5 × 20 s while light was off).

A 5 Group × 15 Session × 2 Phase (CS present, CS absent) ANOVA of nose pokes yielded significant main effects of Group, *F*_(4, 34)_ = 6.12, *p* = 0.001, Session, *F*_(14, 476)_ = 3.42, *p* < 0.001, and Phase, *F*_(1, 34)_ = 20.83, *p* < 0.001, as well as a significant three-way interaction, *F*_(56, 476)_ = 1.56, *p* = 0.008. Panels **(A,B)** of Figure 7 plot the groups' mean nose poke scores for the CS present and CS absent phases, respectively. Comparison of the two panels reveals that the main effect of Phase reflected more overall nose poke responses when the CS was present vs. absent. Therefore, cued responses occurred significantly more often than did pre-mature responses. The main effects of Group and Session were not readily interpreted due to the higher order interaction. The three-way interaction reflected a convergence of scores for the five groups at a relatively stable low level across sessions when the CS was absent [Panel **(B)**], together with a divergence of scores when the CS was present into relatively discrete profiles for each group that paralleled their rank order of reward frequency: from highest (group 100) to lowest (group 25) [Panel **(A)**]. Only the linear trend for the interaction was significant, *F*_(4, 34)_ = 4.03, *p* = 0.009, reflecting the generally consistent increase in nose pokes over sessions in group 100 when the CS was present as against the relatively inconsistent profile of increase in nose pokes across sessions in the other groups during this phase.

#### Habituation to locomotor boxes

A 5 Group × 3 Session ANOVA yielded a main effect of Session, *F*_(2, 70)_ = 23.07, *p* < 0.0001, and no other significant effects, *F*_(8, 70)_ < 1.47, *p* > 0.18. A curvilinear pattern of mean (SE) locomotor scores emerged from session 1, 1076 (74), through session 2, 644 (48), to session 3, 762 (59). Planned comparisons of group 50 with group 0 and with group 100 on the first and final habituation sessions revealed significantly fewer beam breaks in group 50 (*M* = 911; *SE* = 109) vs. group 0 (*M* = 1103; *SE* = 176) on habituation session 1, *t*_(105)_ = 2.02, *p* < 0.05, but no difference between group 50 and group 100 (*M* = 1066; *SE* = 150), *t*_(105)_ < 1.20, *p* > 0.20, on this session. Group 50 did not differ significantly from either group 0 or group 100 on the final habituation session, *t*_(105)_ < 0.93, *p* > 0.30. Therefore, mean drug-free locomotor response in the key groups did not differ consistently prior to testing.

#### Test sessions

**Figure 8:**
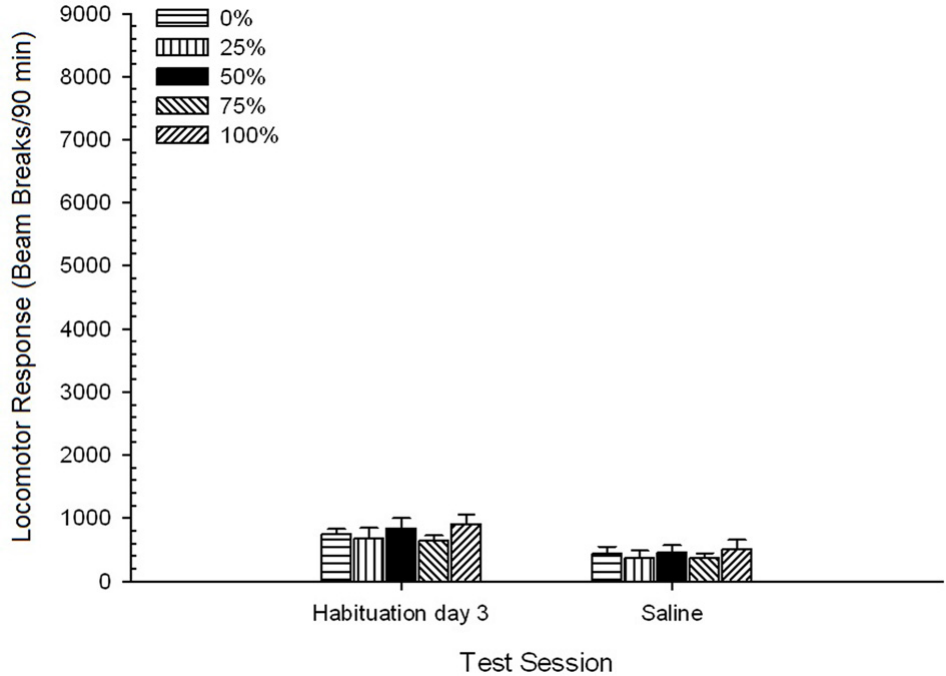
**Mean (SE) locomotor response (number of beam breaks in an electronic array per 90 min) on the last of 3 drug-free habituation sessions and on a subsequent session after saline injection (i.p., 1 ml/kg) in groups of Lewis rats (*n* = 8/group) previously exposed to 15 daily conditioning sessions with sucrose reward (10% solution) delivered under 0, 25, 50, 75, or 100% variable schedules**. The conditioned stimulus was a light (120 s). Group 0 received the same number of rewards as group 100 in the absence of conditioned stimuli.

***Saline***. A 5 Group × 2 Session ANOVA of locomotor responses on the final habituation session and the saline test session yielded a significant main effect of Session, *F*_(1, 35)_ = 50.12, *p* < 0.0001, and no other significant effects, *F*_(4, 35)_ < 0.57, *p* > 0.68. Figure 8 shows the group mean scores for the two sessions and indicates that the Session effect reflected a significant decline from habituation to saline test. Thus, receipt of the injection *per se* (e.g., expectancy, stress) did not enhance locomotor responding.

***Effects of 0.5 mg/kg AMPH***.

*Pre-injection locomotion*. A 5 Group × 2 Session ANOVA of pre-injection locomotion yielded a significant main effect of Session, *F*_(1, 35)_ = 15.04, *p* < 0.001, and no other significant effects, *F*_(4, 35)_ < 1.19, *p* > 0.33. Planned comparisons found no significant difference between group 50 and group 0 or group 100 on either test session, *t*_(70)_ < 0.99, *p* > 0.30. Therefore, baseline differences in pre-injection locomotion did not account for group differences in locomotor response to 0.5 mg/kg AMPH. Mean (SE) beam breaks for the pre-injection phase for the first and second (post-sensitization) 0.5 mg/kg sessions were 325 (25) and 473 (36).

**Figure 9:**
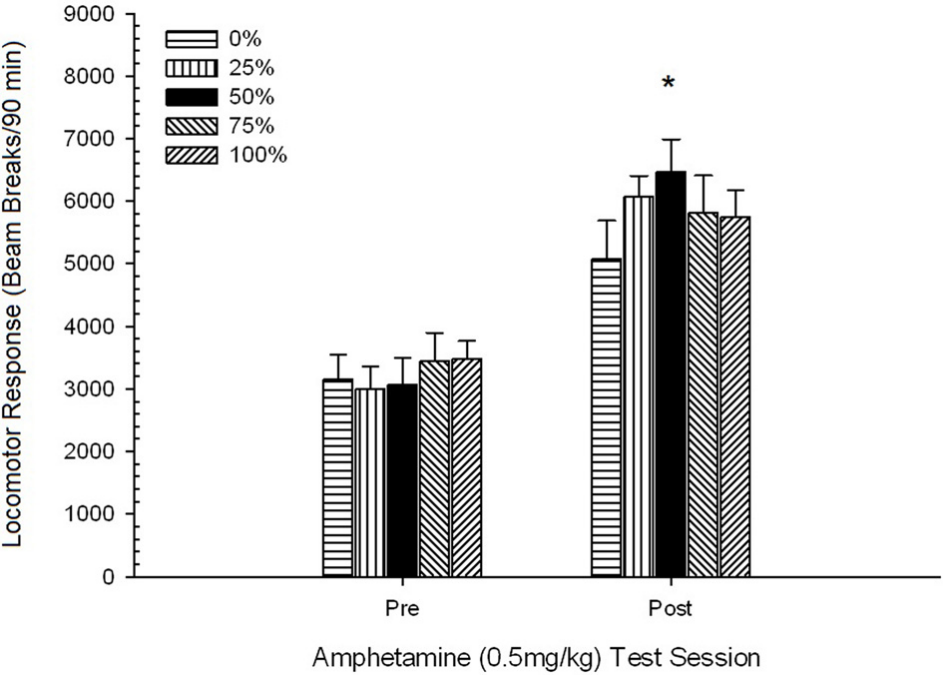
**Mean (SE) locomotor response (number of beam breaks in an electronic array per 90 min) to 0.5 mg/kg d-amphetamine on separate sessions before and after a 5-session sensitizing regimen of d-amphetamine (1.0 mg/kg; i.p. per session) in groups of Lewis rats (*n* = 8/group) previously exposed to 15 daily conditioning sessions with sucrose reward (10% solution) delivered under 0, 25, 50, 75, or 100% variable schedules**. The conditioned stimulus was a light (120 s). Group 0 received the same number of rewards as group 100 in the absence of conditioned stimuli. ^*^p < 0.05 for mean difference between group 50 and group 0 as well as group 100, based on planned comparisons.

*Post-injection locomotion*. A 5 Group × 2 Session ANOVA of locomotor response to 0.5 mg/kg doses delivered before and after chronic 1 mg/kg AMPH yielded a main effect of Session, *F*_(1, 34)_ = 87.44, *p* < 0.0001, and no other significant effects, *F*_(4, 34)_ < 0.94, *p* > 0.45. Figure 9 plots the mean locomotor scores for each group and session and shows that the Session effect reflected an increased overall response to the second 0.5 mg/kg dose, consistent with sensitization. The figure also shows that the groups performed very similarly on session 1, but that group 50 displayed more locomotor activity than the other groups on session 2. Planned comparisons in response to the first 0.5 mg/kg dose revealed no significant differences between group 50 and group 0 or group 100, *t*_(35)_ < 1.28, *p* > 0.20. However, group 50 displayed significantly greater locomotor response to the second 0.5 mg/kg dose than did group 0, *t*_(35)_ = 4.32, *p* < 0.001, or group 100, *t*_(35)_ = 2.24, *p* < 0.05.

***Effects of 1 mg/kg AMPH***.

*Pre-injection locomotion*. A 5 Group × 5 Session ANOVA of 30-min pre-injection scores for the sensitization sessions yielded a main effect of Session, *F*_(4, 140)_ = 4.10, *p* = 0.004, and no other significant effects, *F*_(4, 35)_ = 1.25, *p* > 0.31. Planned comparisons found that beam breaks during the pre-injection phase (M; SE) were significantly lower in group 50 (395; 62) than in group 100 (508; 62), *t*_(175)_ = 2.58, *p* < 0.01, but not group 0, *t*_(175)_ < 1.83, *p* > 0.10, on 1 mg/kg AMPH session 1. On the final 1 mg/kg AMPH session, planned comparisons also found that pre-injection locomotion in group 50 (378; 60) was significantly lower than in group 100 (650; 75), *t*_(175)_ = 6.17, *p* < 0.001, but not in group 0, *t*_(175)_ < 1.84, *p* > 0.10. As the direction of these group differences (control group = group 50) was opposite to the hypothesized pattern, group differences in post-injection locomotion that align with the hypothesis cannot be attributed to pre-injection baseline differences. Mean (SE) overall beam breaks during the pre-injection phase for Sessions 1 through 5 were: 442 (34), 452 (32), 542 (40), 411 (26), 504 (37).

**Figure 10:**
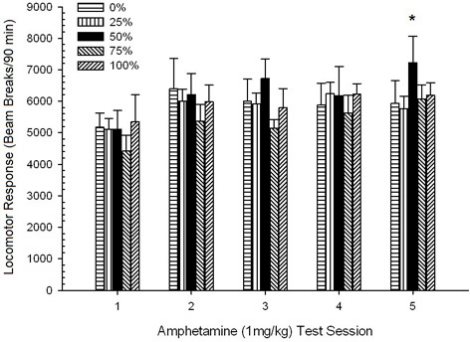
**Mean (SE) locomotor response (number of beam breaks in an electronic array per 90 min) to 1 mg/kg d-amphetamine (i.p.) on 5 weekly sessions in groups of Lewis rats (*n* = 8/group) previously exposed to 15 daily conditioning sessions with sucrose reward (10% solution) delivered under 0, 25, 50, 75, or 100% variable schedules**. The conditioned stimulus was a light (120 s). Group 0 received the same number of rewards as group 100 in the absence of conditioned stimuli. ^*^p < 0.05 for mean difference between group 50 and group 0 as well as group 100, based on planned comparisons.

*Post-injection locomotion*. A 5 Group × 5 Sessions ANOVA of responses to the 1 mg/kg doses yielded a significant main effect of Session, *F*_(4, 140)_ = 6.15, *p* < 0.001, and no other significant effects, *F*_(4, 35)_ < 0.57, *p* > 0.68. Polynomial trend analyses revealed a significant linear trend, *F*_(1, 35)_ = 9.34, *p* = 0.004, and cubic trend, *F*_(1, 35)_ = 5.08, *p* = 0.031, the latter result denoting relative maxima on sessions 3 and 5. Figure 10 plots these scores and shows that, despite the lack of significant interaction in the ANOVA, group 50 exhibited substantially greater locomotion than the other four groups in response to the final 1 mg/kg dose. Accordingly, planned comparisons revealed significantly greater mean scores on session 5 in group 50 than in all other groups, *t*_(35)_ > 3.68, *p* < 0.001.

#### Control for variation in nose poke responding during sucrose training

Two 5 Group × 2 Session ANCOVAs of locomotor response to 0.5 mg/kg AMPH before and after the sensitization regimen, including total nose pokes during sucrose training with CS present and with CS absent as separate covariates, found no significant effects for either covariate, *F*_(1, 32)_ < 0.44 *p* > 0.51. Two 5 Group × 5 Session ANCOVAs of locomotor response to 1 mg/kg AMPH during the sensitization sessions with total nose pokes (CS present, CS absent) as separate covariates yielded no significant effects of the covariate while the CS was present or absent, *F*_(1, 33)_ < 0.14, *p* > 0.71. Therefore, drug-free approach responding did not account for group differences in locomotor responses to either dose of AMPH.

### Discussion

Sensitization developed to the effects of repeated 1.0 mg/kg amphetamine. The habituation and saline data confirm that this effect was not due to pre-existing differences, expectancy, or stress-related responses to the injection. The ANCOVAs with nose pokes confirm that these effects were not due to drug-free approach behavior. The nose poke data themselves indicated that the groups acquired the association between the CS and prospect of sucrose reward. The groups' rank level of nose-poke responding at the end of training matched the overall frequency of reward under the different schedules from highest (group 100) to lowest (group 0), as it did in experiment 2. The relatively lower overall mean nose poke levels in this experiment compared to experiments 1 and 2 may reflect more selective approach responding to cues for reward in Lewis rats (Kosten et al., 2007).

The 0.5 mg/kg dose data showed that initial locomotor response to AMPH in Lewis rats (Figure 9) was somewhat suppressed compared to Sprague Dawley rats (experiment 2; Figure 5), but the within-group increase in response to the second dose in Lewis rats was considerable (nearly double the response to the first 0.5 mg/kg dose) following the 5-session AMPH regimen Most notably, group 50 displayed a greater locomotor response than all groups except group 25 to the second (i.e., post-sensitization) 0.5 mg/kg AMPH dose and a greater locomotor response than all other groups, including group 25, to the final 1 mg/kg AMPH dose (final sensitization session).

#### Summary analysis of group rankings across experiments

To determine the reliability of group differences in sensitization, a non-parametric analysis assessed the contingency between group and rank of mean locomotor response to the second (post-chronic AMPH) 0.5 mg/kg dose and the final 1.0 mg/kg dose of AMPH from the 3 experiments. The analysis yielded a significant effect, φ = 0.986, *p* = 0.025, reflecting the fact that group 50 ranked first in all but one of the comparisons. The superior rank of group 50 compared to all other groups in response to the second (post-chronic AMPH) 0.5 mg/kg dose is depicted in Figure 5 (experiment 2) and Figure 9 (experiment 3). The superior rank of group 50 relative to other groups in response to the final 1.0 mg/kg dose is depicted in Figure 2 (experiment 1) and Figure 10 (experiment 3). The only exception to this pattern was the response to the final 1.0 mg/kg dose in Sprague-Dawley rats in experiment 2.

## General discussion

The present series of experiments tested the hypothesis that chronic exposure to a gambling-like schedule of reward can sensitize brain DA pathways much like chronic exposure to drugs of abuse. Evidence for such an effect would suggest that neuroplasticity, of the same kind thought to contribute to drug addiction, can be induced by chronic exposure to unpredictable reward schedules. In line with the literature on drug addiction, locomotor response to 0.5 and 1.0 mg/kg doses of AMPH indexed DA system reactivity, with greater locomotion in response to later doses operationally defining sensitization (cf. Robinson and Berridge, 1993; Pierce and Kalivas, 1997; Vanderschuren and Kalivas, 2000).

Overall, the results are in line with our hypothesis. However, they also indicate considerable variability in experimental effects due to procedural factors. The effects of conditioning schedule were modest but consistent, with group 50 demonstrating greater response than the other four groups to both doses following the five dose-regimen. Although overall *F*-values for group-related effects in the variance analyses were often non-significant, key group differences were confirmed with pairwise planned comparisons. In this regard it should be noted that, “Current thinking, however, is that overall significance [for *F* in the ANOVA] is not necessary. First of all, the hypotheses tested by the overall test and a multiple-comparison test are quite different, with quite different levels of power. For example, the overall *F* actually distributes differences among groups across the number of degrees of freedom for groups. This has the effect of diluting the overall *F* in the situation where several group means are equal to each other but different from some other mean” (Howell, 1992, p. 338). This is the precisely the situation that applied in the present experiments, where group 50 was expected to differ from group 0 and group 100 controls but no difference between these control groups was predicted for group 25 or group 75.

The nose poke data confirmed that, in every experiment, the animals acquired the association between the CS and the prospect of sucrose reward. The correspondence between nose poke frequency for the different groups and overall frequency of reward under their respective training schedules suggests that the average rate of sucrose reward guided drug-free approach responding. However, the lack of mediating effect of nose pokes on group-related locomotor responses to AMPH in the ANCOVAs indicated that separate processes underlie the two behaviors.

In some cases, the effect of conditioning schedule was evident in response to the first AMPH dose; in other cases it only emerged after repeated doses. Group differences in locomotor response to the first AMPH dose suggest that exposure to gambling-like reward schedules is sufficient by itself to induce sensitization. Group differences in locomotion following multiple AMPH doses indicate a more subtle effect that could be characterized as “susceptibility,” which only manifests when combined with ongoing exposure to the primary sensitizing agent (i.e., amphetamine).

Differences in the pattern of response across experiments suggest that a longer interval between training and initial AMPH challenge may maximize the opportunity to detect the inherent sensitizing effect of the conditioning treatment. This in turn suggests that effects of conditioned reward exposure may incubate over time, a phenomenon also seen with stimulant sensitization (Grimm et al., 2006). The pattern of response to the two doses of amphetamine suggests that the 0.5 mg/kg dose may be more effective in revealing the effects of conditioning history. This in turn suggests that conditioning effects under the current training protocol are somewhat subtle and may be camouflaged by ceiling effects under doses of AMPH and conditions that generate *de novo* sensitization.

In experiment 3, the biphasic pattern of response to the 0.5 mg/kg doses and progressive emergence of superiority in group 50 is consistent with the expected profile for Lewis rats in response to methamphetamine (Camp et al., 1994). This lends support to the validity of the present findings and suggests overlap between the factors that moderate vulnerability to psychostimulant sensitization and to gambling-like schedules of reward.

Across experiments, the post-sensitization locomotor response of group 50 generally exceeded that of the other groups under different doses of amphetamine and in different strains of animals. However, the high within-group variability and modest between-group effect sizes indicate a role for other factors in DA system reactivity to amphetamine following exposure to varying schedules of conditioned sucrose reward. Although responses of DA neurons to reward signals may provide a coarse model of gambling (Fiorillo et al., 2003), like all models, there is a loss of information for the sake of parsimony—i.e., to demonstrate a key process. As a result, the pattern of effects across CS-US conditions in the original Fiorillo et al. study does not fully generalize to locomotor response to amphetamine. Further refinements of the model are called for to fully capture the aspects of gambling that impact on DA system function.

Taken together, the results of this series of experiments provide provisional support for the hypothesis that chronic exposure to gambling-like schedules of reward enhances the reactivity of the brain DA system to psychostimulant challenge. As such, they extend the findings of Singer et al. (2012) who demonstrated that, relative to a fixed schedule, prior exposure to a variable reinforcement schedule in an operant paradigm enhances subsequent locomotor response to amphetamine. More specifically, the present findings point to uncertainty of reward delivery as the critical factor underlying the effects of variable reward. The magnitude of effects in the operant paradigm was substantially greater than the effects found in the present experiments. This may reflect greater chronic exposure to the gambling-like activity (55 vs. 15 days); it may reflect the effects of requiring an operant response to elicit the reward (i.e., a role for agency) rather than passive exposure, as in the present study. Increasing the duration of training in the present paradigm would help to resolve these questions.

The validity of variable reward and reinforcement schedules as models of gambling cannot be gleaned from these experiments. Future research that examines the impact of conditioning history on risk-taking behavior in rodent gambling tasks could address this issue. Similarly, the correspondence between the behavioral sensitization found here and the elevated striatal DA response to amphetamine recently found in pathological gamblers must await further investigation (Boileau et al., 2013). Micro-dialysis could address this question, and the prediction based on the human data would be that greater DA release in the group 50 “gambling phenotype” would be most clearly observed in the dorsal (sensorimotor) striatum rather than the ventral (limbic) striatum. Validation of 50% variable CS + reward exposure in these other paradigms would support its utility as a bona fide experimental model of PG.

Whereas some forms of gambling clearly entail an instrumental response (e.g., slot machines), in other forms of gambling (e.g., lottery) the link between the action (purchasing the ticket, i.e., placing the bet), the cues for reward (i.e., lottery numbers) and the reward itself (the winning number and monetary payoff) is much more diffuse. Nevertheless, activation of DA during the CS-US interval may well occur. This may explain why, when the “winning number” is announced, attention is riveted as each individual lottery ball drops in succession to compose the specific sequence of digits in the winning number. Although the probability of a specific digit occurring is mathematically defined, the outcome for each individual lottery ball is binary—hit (matches the player's number) or miss (does not match the player's number)—and the outcome on any given trial is unknown. Such a scenario may better characterize the experience of group 50 in the present experiments, where reward was provided non-contingently but also unpredictably and the CS merely indicated the potential for reward without revealing whether it would occur on a given trial. Slot machines are more strongly linked with PG than are lottery tickets (Cox et al., 2000; Bakken et al., 2009), indicating an important role for instrumental factors (and immediacy) in the rewarding aspects of gambling for this population (Loba et al., 2001). Nonetheless, the Pavlovian process modeled in the present experiments (CS + uncertain reward) appears to be a necessary if not sufficient element of the gambling experience.

Along with the lack of a clear instrumental requirement, a number of other design features may have contributed to the relatively modest and variable pattern of experimental effects. The groups differed in overall sucrose exposure as well as the contingency between CS and sucrose reward. Although this may have contributed to inter-group variability, it cannot readily explain why animals with the greatest sucrose exposure (group 100) displayed less sensitization than group 50. In addition, group 0 received no stimulus before sucrose exposure on every trial. Although this precluded a cue-induced expectation of reward, it did not control for the presence of a stimulus before reward delivery, which existed in all other groups. To address this issue, future research should include a condition where animals receive reward on every trial following random exposure to a neutral stimulus (i.e., whose presence does not signal the potential for reward).

Another design limitation is the potential emergence of adjunctive behavior that could influence the effects of training schedule. In the face of uncertainty, animals may develop superstitious behaviors designed to enhance perceived control and reduce uncertainty-induced DA activation (cf. Harris et al., 2013). It is therefore possible that uncontrolled aspects of the experimental design enabled the animals to offset the effects of conditioning schedule. Such an effect could contribute to the relatively modest and variable response to amphetamine in group 50 following CS + sucrose training. Future research should record spontaneous behavior, aside from nose pokes, during training sessions to test this possibility, and control for it statistically should it emerge. Because such behavior would be expected to counteract or dampen the effects of schedule-induced uncertainty, locomotor response to amphetamine in group 50 should be enhanced when it is controlled (procedurally or statistically). Therefore, the present (uncontrolled) design provides a conservative test of the effects of 50% CS + reward on amphetamine sensitization.

In terms of external validity, the use of male rats also limits the generalizability of the results. The lack of a clear “punishment” condition also differs from gambling, where large monetary losses are common and exert important motivational effects (Nieuwenhuis et al., 2005; Singh and Khan, 2012). The ability to accumulate reward is also absent from the present paradigm and cumulative winnings in a slot machine game have been found to interact with DA manipulations in humans (Tremblay et al., 2011; Smart et al., 2013). Similarly, the opportunity for a jackpot is an important difference between the present model and actual gambling.

Despite these limitations, the present results suggest that 50% variable CS + reward can engage DA pathways implicated in the reinforcing effects of gambling (Fiorillo et al., 2003; Anselme, 2013). Cross-sensitization of response to AMPH following this gambling-like schedule is consistent with a pivotal role for DA in gambling and psychostimulant drug effects (Zack and Poulos, 2009), and extends earlier studies on cross-priming of motivation to gamble by AMPH in pathological gamblers (Zack and Poulos, 2004). The present results also indirectly suggest that modest doses of AMPH, which do not cause supra-physiological DA release, may better model brain activity in response to intermittent reward signals (i.e., during gambling) than exposure to high (i.e., binge-like) doses of stimulant drugs (cf. Vanderschuren and Pierce, 2010). Direct support for this correspondence could be derived by assessing DA release in response to the 50% variable CS-US schedule and different doses of AMPH using microdialysis.

From an experimental standpoint, the present Pavlovian model and the previous operant model of variable reinforcement both appear to engender a phenotype resembling the human pathological gambler. As such, they provide a valuable complement to rodent gambling tasks which model gambling behavior (as a dependent measure) but have, until now, only employed healthy animals, the equivalent of human social gamblers. Based on the literature, the animals chronically exposed to variable reward may well differ in these tasks, particularly in response to DA-ergic drugs. Combining the rat gambling phenotype with gambling tasks may permit systematic development of medications for the treatment of PG, which might not be fully accomplished with healthy animals alone. Further refinements in the experimental design and training regimen, as described above, should improve the correspondence between animals trained in this paradigm and actual pathological gamblers.

From the clinical-sociological standpoint, the finding that exposure to 50% variable CS + reward, which closely matches the reward schedule on a commercial slot machine (Tremblay et al., 2011), changes the brain DA system in reliable and enduring ways suggests that, in some cases, gambling activity, like drugs of abuse, may be a “pathogen” capable of causing addiction. However, the modest effect size and high variability in response to 50% CS + reward suggest that, like drugs of abuse, the tendency for gambling-like reward schedules to promote addiction will depend greatly on the pre-existing risk profile of the gambler. Nevertheless, to spare high risk individuals exposure to potential adverse gambling-related effects, it seems reasonable that policies applied to deter use and minimize harm from drugs of abuse could be extended to gambling as well.

### Conflict of interest statement

The authors declare that the research was conducted in the absence of any commercial or financial relationships that could be construed as a potential conflict of interest.
